# Bacterial outer membrane vesicles OMV-LL for delivery of neoantigen mRNA to induce anti-HCC therapy

**DOI:** 10.3389/fimmu.2025.1633345

**Published:** 2025-08-29

**Authors:** Jiaqing Cheng, Suxin Wu, Chenlu Zhu, Shengzhe Lin, Fang Liu, Shuping Chen, Yunbin Ye

**Affiliations:** ^1^ The School of Basic Medical Sciences, Fujian Medical University, Fuzhou, Fujian, China; ^2^ Laboratory of Immuno-Oncology, Clinical Oncology School of Fujian Medical University, Fujian Cancer Hospital, Fuzhou, Fujian, China; ^3^ Department of Hepatobiliary Surgery and Fujian Institute of Hepatobiliary Surgery, Fujian Medical University Union Hospital, Fuzhou, Fujian, China; ^4^ Key Laboratory of Translational Cancer Medicine, Fuzhou, Fujian, China

**Keywords:** mRNA, TP53, cytotoxic T lymphocytes, outer-membrane vesicles, hepatocellular carcinoma

## Abstract

**Introduction:**

Hepatocellular carcinoma (HCC) represents a significant health challenge, with immunotherapy serving as a crucial component of its complex treatment regimen. This study investigates the use of TP53Y220C as a preferred antigen to induce cytotoxic T lymphocytes (CTLs) for cytotoxic effects against HCC.

**Methods:**

The TP53_Y220C_ mRNA (mTP53_Y220C_) was synthesized through an *in vitro* transcription method and subsequently introduced into dendritic cells (DCs) using bacterial outer membrane vesicles expressing L7Ae and Listeria monocytogenes lysin O (OMV-LL), electroporation, and lipid nanoparticles, respectively. Co-culture of differently treated DCs with initial T cells induces CTLs. The cytotoxic effects of CTLs on hepatocellular carcinoma were evaluated through experiments such as flow cytometry and mouse tumour models.

**Results:**

We assessed the therapeutic efficacy of CTLs, activated by mTP53_Y220C_-loaded DCs, in a murine model of HCC. Results demonstrate that CTLs, activated by DCs loaded with mTP53_Y220C_ via OMV-LL or electroporation, effectively initiated immune responses against HCC. While OMV-LL were less efficient than electroporation in mRNA delivery, they induced a significant pro-inflammatory response and activated the innate immune system.

**Conclusion:**

This study highlights OMV-LL as an innovative mRNA delivery approach to DCs for CTLs activation and demonstrates their potential in CTLs-based therapy for HCC.

## 1 Introduction

According to data from the National Cancer Center of China, by 2022, liver cancer had the fourth-highest incidence and the second-highest mortality rate, representing a significant public health burden in China (1). The multidisciplinary treatment of hepatocellular carcinoma (HCC), particularly immunotherapy, merits exploration.

Anticancer vaccines, classified as therapeutic (2), have undergone numerous clinical trials to evaluate their tumor-antigen targeting efficacy. These trials have yielded promising results that support their practical applications (3, 4), Tumor antigens, categorized into TAA and TSA, are crucial in cancer immunotherapy. Being exclusively present in cancerous cells, TSA is particularly suitable for targeted immunological interventions (5), of which neoantigens arise from non-synonymous mutations and receive significant attention. These include personalized variants, unique to individual patients, and shared variants, common across various tumor types or among different patients. Each patient exhibits a unique neoantigen profile, necessitating whole exome sequencing of the patient’s cancer and adjacent tissues to identify and screen for individual neoantigens. However, this process is time-consuming and costly, limiting its practical application. Consequently, therapies based on shared neoantigens, expressed in a broader patient population, are more feasible for widespread use (5, 6).

We focused here on TP53, a crucial oncogene in tumorigenesis and progression, which is mutated in approximately 50% of all cancer types, with the highest mutation frequency observed in hepatocellular carcinoma (7–9). The TP53 mutation is strongly associated with poor prognosis in advanced malignancies, making it a prime target for cancer therapies (9, 10). The shared neoantigen TP53_Y220C_, frequently found in HCC through database screening and extensive literature reviews, has been identified as a potential vaccine target.

The landscape of neoantigen vaccines is diverse, encompassing peptides, mRNA, and DNA forms. Among these, mRNA vaccines offer distinct advantages, such as preventing genomic integration, avoiding mutagenesis, being non-persistent in the body, thus reducing the risk of prolonged side effects, and their capacity to encode multiple antigens, facilitating tailored treatment plans and complex therapeutic strategies (11).

However, the hydrophilic nature and negative charge of mRNA molecules make them susceptible to degradation and instability and pose challenges for cellular uptake, thus limiting their bioavailability *in vivo*. Consequently, numerous studies have focused on enhancing the stability of mRNA and developing efficient delivery systems. Although improvements to mRNA architecture, including the addition of a 5′ cap and a 3′ poly-A tail, have significantly increased its stability and translational efficacy (12–14), chemical modification of mRNAs, such as the use of nucleotide analogs pseudouridine to reduce immunostimulatory effects, is another important way to improve the efficiency and stability of mRNA translation (15). Moreover, mRNAs belong to large molecules with a negative charge, and it is difficult to enter the cell through the negatively charged lipid bilayer of the cell membrane. Hence, the efficiency of cellular loading of mRNA is also critical for vaccine efficacy. Beyond traditional viral carriers, innovative delivery systems such as lipid nanoparticles, extracellular vesicles, and electroporation have shown promising results in clinical studies (16–18). Among them, electroporation is a frequently used mRNA delivery method for the preparation of DC vaccines in clinical trials. However, this requires the availability of large-scale clinical-grade electroporation equipment, in addition to involving the *in vitro* isolation and culture of human cells. This process is expensive and prone to contamination, so there is a need to find an alternative, readily available, inexpensive, and alternative delivery method.

This research assessed the comparative efficacy of CTLs stimulated by DC vaccines created through various mRNA transfection techniques. We employed several delivery methods to transfect neoantigen mRNA into DCs, including liposomes, bacterial outer membrane vesicles (OMVs), and electroporation, to evaluate their effectiveness in activating CTLs and inducing cytotoxicity. This study investigates the differences in the anti-tumor effects of CTLs induced by DC vaccines generated through different delivery modalities, offering a critical strategy for the further optimization of cancer immunotherapy.

## 2 Materials and methods

### 2.1 Cell lines

The hepatocellular carcinoma cell lines Huh-7 endogenously expressing TP53_Y220C_ and SK-Hep-1, obtained from the cell bank of the Shanghai Institute for Biological Sciences, Chinese Academy of Sciences (Shanghai, China), were routinely screened for mycoplasma to ensure purity. Additionally, considering that CTLs are HLA-restricted in recognizing target cells for killing effects, a target cell line Huh7-A0201, stably engineered to express HLA-A*02:01, was constructed. The Huh-7 and Huh7-A0201 cell lines were cultured in Dulbecco’s Modified Eagle’s Medium, procured from BasalMedia (Shanghai, China). Meanwhile, SK-Hep-1 cells were cultured in Minimum Essential Medium, also sourced from BasalMedia. Both media were supplemented with 10% fetal bovine serum from PAN-Seratech GmbH (Aidenbach, Germany) and 1% penicillin-streptomycin from MP Biomedicals (California, USA). The cells were incubated at 37°C in a 5% CO2 atmosphere.

### 2.2 Animals

To further evaluate the killing effect of human-derived CTLs on hepatocellular carcinoma *in vivo*, immunocompromised NOD/SCID mice were selected as animal models with being inoculated human HCC cell lines subcutaneously, and peritumoral injection of CTLs for assessing its inhibitory effect on hepatocellular carcinoma. Male NOD/SCID mice, aged 4 to 5 weeks, were sourced from Guangdong Yaokang Biotechnology Co., Ltd. (Foshan, China). They were housed in a Specific Pathogen-Free facility at the Laboratory Animal Center of Fujian Medical University (Fuzhou, China), with protocols approved by the university’s Animal Care and Use Committee. The experimental procedures involving these mice strictly adhered to the established guidelines for animal welfare.

### 2.3 Generation of monocyte-derived DCs

Monocytes were isolated from the peripheral blood of healthy individuals, followed by the separation of CD14^+^ monocytes using magnetic bead technology. These cells were cultured in a Lymphocyte Serum-free Medium supplied by Corning Incorporated. Cultivation occurred in six-well plates, supplemented with 100 ng/mL GM-CSF and 50 ng/mL IL-4, both sourced from MCE Tech. Cytokines were replenished on the third and fifth days, and the cells were harvested on the seventh day for experimental use. This research was approved by the Ethical Review Board of Fujian Cancer Hospital under the reference number K2023-455-01. Blood samples were obtained from consenting volunteers who had signed informed consent documents.

### 2.4 Generation and isolation of OMV-LLs

To obtain a kind of extracellular vesicle to deliver mRNA, chimeric genes encoding ClyA fused to L7Ae or LLO were constructed and inserted into the multiple cloning sites of the pACYCDuet-1 vector, respectively. Plasmids encoding the RNA-binding protein L7Ae and the lysosomal escape protein LLO were recombinantly produced and introduced into Escherichia coli BL21(DE3) for expression. Positive clones were selected on chloramphenicol plates (50 µg/mL) and cultured in an LB medium for mass amplification. Initially, the liquid portion of the bacterial culture was collected, and a 10-min centrifugation at 4,000 g was performed to remove the bacteria. This was followed by a secondary centrifugation at 24,000 g for 3 h to isolate the outer membrane vesicles (OMV-LL).(19).

### 2.5 Acquisition and purification of neoantigen mRNA

The pcDNA3.1(+) plasmid was engineered to include the following sequences after the T7 promoter: alpha-globin 5’UTR, KOZAK sequence, SP sequence (MHC class I signaling peptide designed to guide the peptide through the endoplasmic reticulum into the lumen), TP53_Y220C_ sequence (GTGGTGCCCTGTGAGCCGCCTGAGGTT), intermediate linker (GGSGGGGGSGG), TP53_Y220C_ sequence, intermediate linker, FLAG tag, and alpha-globin 3’UTR. mRNA encoding neoantigens was synthesized via *in vitro* transcription using T7 polymerase. The process began with linearization of the plasmid DNA by the XbaI restriction enzyme, which then served as the template for the transcription reaction facilitated by a Transcription Kit from Thermo Fisher Scientific (MA, USA). Following transcription, the mRNA was purified using a Transcription Cleaning Kit from the same manufacturer, adhering to their provided protocol.

### 2.6 Evaluation of mRNA transfection efficiency

DCs (1×10^5^ cells per well) without any treatment were used as the blank control group. DCs (1×10^5^ cells/well) were seeded into 24-well plates 24 h before transfection. For transfection, 9 μL of OMV-LL and 1.5 μL of Lipofectamine™ 3000 were mixed with 1 μL of mRNA encoding EGFP (mEGFP, APExBIO Technology LLC, USA) in opti-MEM medium for 15 min; the mixture was then added to the cells in a 24-well plate. 24 hours later, fluorescence images of each culture well were captured under microscope to assess transfection efficiency, and quantitative analysis was performed using flow cytometry. For electroporation, 1×10^6^ cells were collected, resuspended in 100 μL of opti-MEM medium, and transferred into a 2 mm electroporation cuvette (Suzhou Etta Biotech, China). The cuvette was placed in the X-Porator H1 electroporator (Suzhou Etta Biotech, China) and subjected to electroporation under the following conditions: 200 V voltage, 3 pulses, 800 µs duration, and 612 ms interval. Afterward, 1×10^5^ cells were removed and seeded into 24-well plates. 24 hours later, images of each well were captured using a microscope to assess transfection efficiency, which was quantitatively analyzed through flow cytometry.

### 2.7 Western blot analysis

Proteins from lysed DCs were isolated and analyzed via Western blot. The primary antibodies used were Mouse anti-DDDDK-Tag mAb and Mouse anti-Human β-actin mAb, both diluted according to the recommendations provided by the supplier. These were followed by horseradish peroxidase-conjugated secondary antibodies. Detection was performed using the enhanced chemiluminescence (ECL) method, following Pierce’s protocol for the ECL kit. The antibodies were sourced from ABclonal Technology Co., Ltd. (Wuhan, China).

### 2.8 Enzyme-linked immunosorbent assay

ELISA plates were prepared by coating them with either IFN-γ or TNF-α capture antibodies and incubating them overnight at 4°C. Subsequently, the plates were washed with a washing solution and blocked with a blocking solution for 4 h at room temperature. Samples were then added to the plates and incubated overnight at 4°C. After another washing step, the plates were incubated with secondary antibodies for 1 h at room temperature. This was followed by a 30-min incubation with streptavidin-HRP at room temperature. After a final wash, the plates were developed with TMB for 15 min at room temperature. The color development was stopped using a stop solution, and the absorbance was measured at 450 nm.

### 2.9 Enzyme-linked immunospot assay

The Human IFN-γ ELISpot kit was procured from Dakewe Biotech (Shenzhen, China). Following the kit’s instructions, the plates pre-coated with an antibody specific to IFN-γ were rinsed with PBS and then incubated with RPMI 1640 medium supplemented with 10% FBS for 10 min at room temperature. After removing the RPMI 1640 medium, CTLs (5×10^4^ cells/well) and tumor cells (1×10^4^ cells/well) were added to the plates. The plates were then cultured at 37°C for 24 h. Post-washing with PBS, the plates were incubated with biotinylated IFN-γ antibody and streptavidin-HRP, each for 1 h at 37°C. The color development was performed using AEC at room temperature in the dark for 30 min. After washing, the plates were air-dried in the dark, and the spots were counted using an ELISpot reader (CTLS6).

### 2.10 Tumor-specific CTL killing assay

Target cells (Huh7-A0201 or SK-Hep-1) were cultured with CTLs at different E/T ratios in 96-well plates at 37°C for 6h. The Cytotoxicity Assay kit (Promega, Madison, WI, USA) was used to measure LDH activity in the supernatant, indicating cell damage. The cytotoxic cell percentage was determined by the formula: (Cocultivation LDH release - Tumor spontaneous release - CTLs spontaneous release)/(Tumor maximum release - Tumor spontaneous release) × 100%, which evaluates the cytotoxic effect of CTLs on the target cells.

### 2.11 Flow cytometric analysis

Flow cytometric analysis was conducted using a FACS Canto Ⅱ flow cytometer from BD Biosciences (CA, USA). The antibodies utilized in the flow cytometry analysis included anti-CD80 labeled with FITC, anti-CD86 labeled with PE, anti-CD137 labeled with PE, anti-CD28 labeled with PE, and anti-CD69 labeled with FITC, all of which were sourced from BD Pharmingen. Data analysis was carried out with FlowJo version 10 software.

### 2.12 Immunization and tumor challenge

Male NOD/SCID mice were subcutaneously inoculated with Huh7-A0201 cells (1×10^6^ cells/spot). On day 7 post-inoculation, CTLs (2×10^6^ cells/spot) were injected peri-tumorally. Tumor sizes were monitored every two days using calipers.


Tumor volume=longest dimension×shortest dimension22

### 2.13 Histological analysis

Tissues from the heart, liver, spleen, lungs, and kidneys were extracted from the mice and fixed in paraffin. These tissue sections were then subjected to dewaxing, rehydration, and staining using Mayer’s hematoxylin and eosin staining method.

### 2.14 Statistical analysis

Data are presented as the mean ± standard deviation, except where specified otherwise. Statistical analysis was conducted using GraphPad Prism version 8.0. Significance was determined using either an unpaired t-test or a one-way ANOVA. The levels of significance were denoted as follows: ns (no significant difference); *p<0.05; **p<0.01***p<0.001.

## 3 Results

### 3.1 Identification of neoantigens and construction of mRNA delivery system

**Figure 1:**
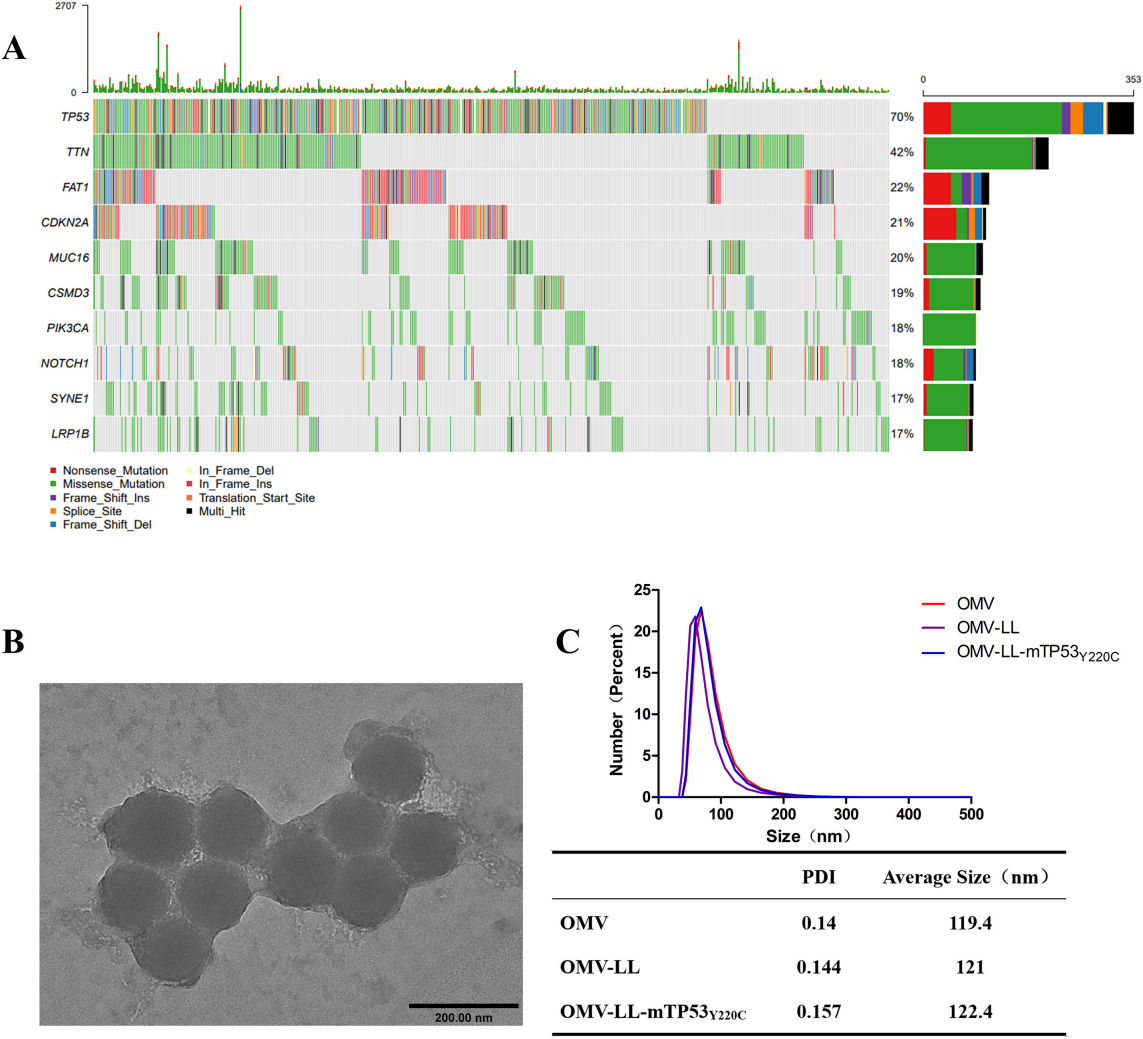
Screening for hepatocellular carcinoma neoantigens. **(A)** Top ten mutated genes in 502 liver cancer samples. **(B)** Transmission electron microscopy diagram of OMV-LL (scale bar: 200 nm). **(C)** Dynamic light scattering detection of nanoparticle size and polydispersity index.

The dataset comprising 502 hepatocellular carcinoma (HCC) cases was sourced from the cBioPortal website and analyzed for mutation characteristics using the “Maftools” R package developed by Mayakonda (20). Mutation types included non-synonymous changes, premature stop codons, frameshift and non-frameshift deletions, and insertions, both with and without frameshift. Out of 502 samples, 458 mutations were detected. The ten genes exhibiting the highest mutation rates were identified as TP53 TTN, FAT1, CDKN2A, MUC16, CSMD3, PIK3CA, NOTCH1, SYNE1, and LRP1B, with respective mutation rates of 70%, 42%, 22%, 21%, 20%, 19%, 18%, 18%, 17%, and 17% (**Figure 1A**). Consequently, TP53 was selected for further study in anti-HCC immunotherapy due to TP53_Y220C_, a relatively common mutation in HCC, serving as the focus of our study (21, 22). The HCC cell line Huh-7, harboring the endogenous TP53_Y220C_ mutation, was identified using the TP53 Database website. Huh7-A0201, transfected with HLA-A*02:01, was utilized as a specific target cell, while SK-Hep-1, also expressing HLA-A*02:01 but lacking the TP53_Y220C_ mutation, served as a non-specific target cell.

The OMV-LL presents the RNA-binding protein L7Ae and the lysosome-escaping protein LLO on its surface (19). The OMV-LL vector was linked to the box C/D sequence of mRNA by L7Ae and achieved lysosomal escape of antigen via LLO. Verification of OMV-LL dimensions through dynamic light scattering and electron microscopy revealed a consistent size of around 120 nm. (**Figures 1B, C**).

### 3.2 OMV-LL efficiently delivers antigen and induces CTLs activation

**Figure 2:**
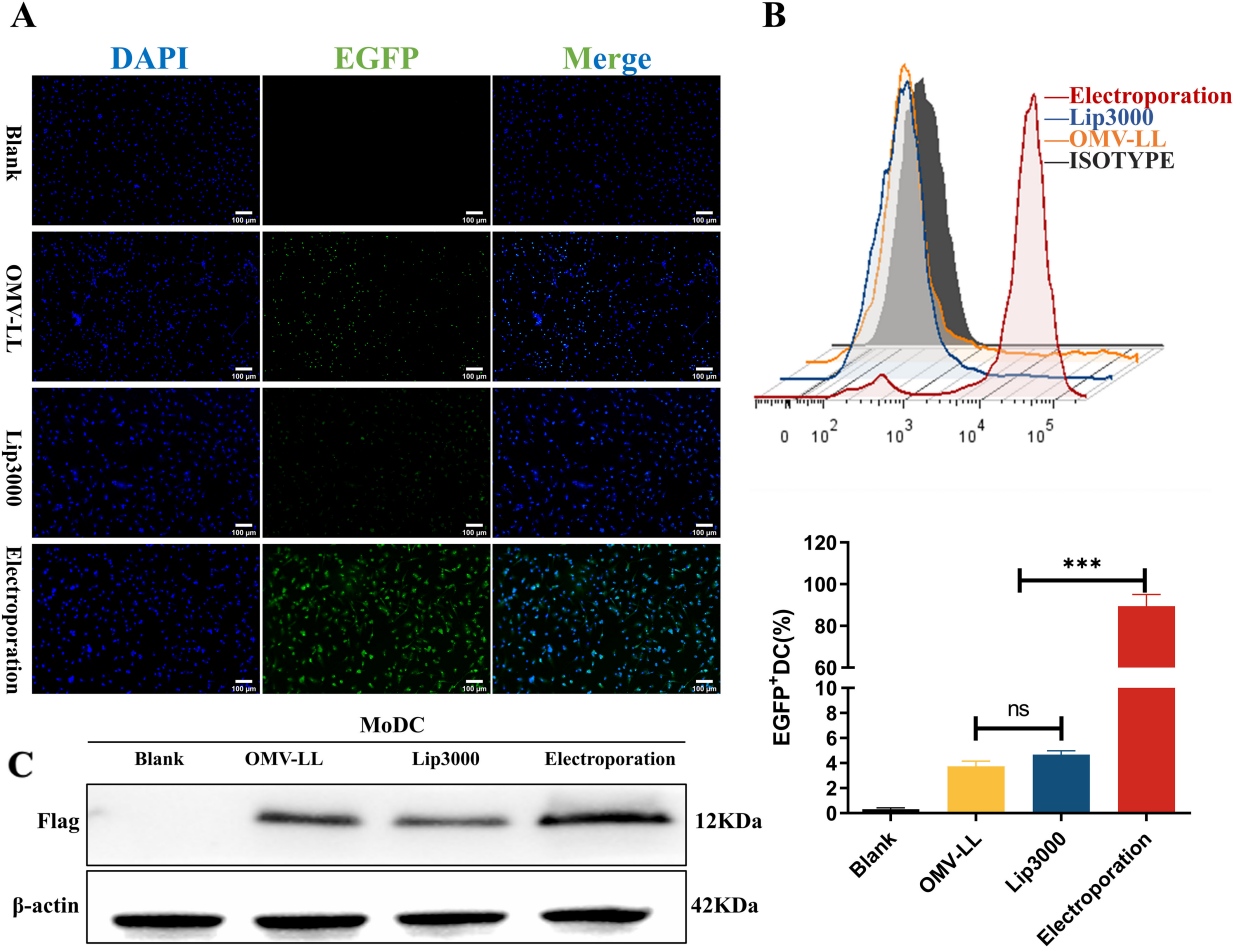
Comparison of DC transfection efficiency among three mRNA delivery modes. **(A)** Fluorescence microscopy to observe the expression of green fluorescent protein in DCs (scale bar: 100µm). **(B)** Flow cytometry was performed to detect the percentage of EGFP^+^ DCs and statistically analyzed. **(C)** Western Blot to detect TP53_Y220C_-Flag fusion protein expression. (***P<0.001).

Monocyte-derived dendritic cells (MoDCs) were transfected with mEGFP using various delivery methods. The success of the transfection was evaluated by observing the expression of green fluorescent protein under a fluorescence microscope and quantifying the proportion of EGFP^+^DCs via flow cytometry. Compared with the blank control group, effective mRNA delivery was observed in the other three groups (**Figure 2A**). The transfection efficiency of the OMV-LL delivery system was approximately 3.75 ± 0.57%, that of the Lip3000 lipid nanoparticles was approximately 4.67 ± 0.43%, while the electroporation method achieved a significantly higher efficiency of approximately 89.47 ± 7.88%, markedly surpassing the performance of both OMV-LL and Lip3000 (**Figure 2B**).

MoDCs were transfected with mRNA encoding the mTP53_Y220C_-Flag for further validation using the same delivery methods. Western blot analysis showed higher expression levels of the TP53_Y220C_-Flag fusion protein in the electroporation group compared to the two groups (**Figure 2C**).

**Figure 3:**
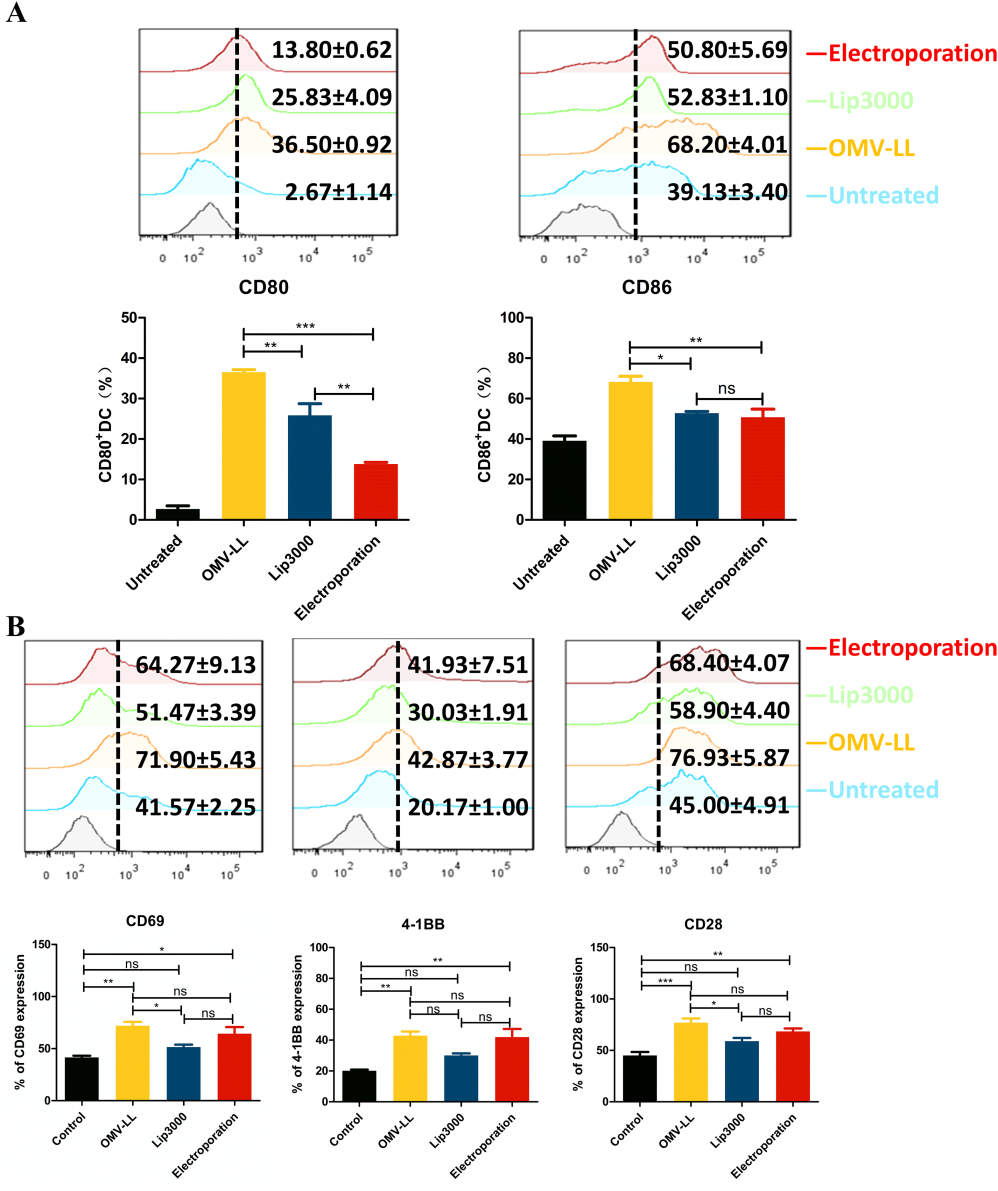
Different transfection modes load neoantigen mRNA to promote DCs maturation and CTLs activation. **(A)** Expression of CD80 and CD86 on the surface of DCs transfected with the three delivery modes was detected by flow cytometry and statistically analyzed. **(B)** Flow cytometry was used to detect the expression of CD69, a CTL surface activation indicator, and CD28 and CD137, co-stimulatory molecules in each group, and statistical analysis was done. (*P<0.05, **P<0.01, ***P<0.001).

However, while investigating the impact of three different mRNA delivery methods on the maturation of DCs, it was found that all three delivery approaches significantly increased the expression levels of CD80 and CD86 on DC surfaces compared to the untreated control group. Notably, the OMV-LL group demonstrated significantly higher expression levels than the Lip3000 and electroporation groups (**Figure 3A**). These results implied that OMV-LL could induce a highly efficient immune response, although its efficiency in delivering mRNA is limited.

Additionally, the expression levels of CD69, a surface indicating cellular activation, along with the co-stimulatory molecules CD28 and CD137, were assessed via flow cytometry. The results showed a substantial increase in the expression of CD69, CD28, and CD137 on the surfaces of CTLs in both the OMV-LL and electroporation groups compared to the control group (**Figure 3B**). This suggests that introducing mTP53_Y220C_ into DCs using OMV-LL and electroporation methods more effectively promotes T-cell activation, thereby enhancing the anti-tumor effects of the subsequent CTL responses.

### 3.3 CTLs induced by mTP53_Y220C_ via OMV-LL obtained powerful anti-tumor effect *in vitro*

**Figure 4:**
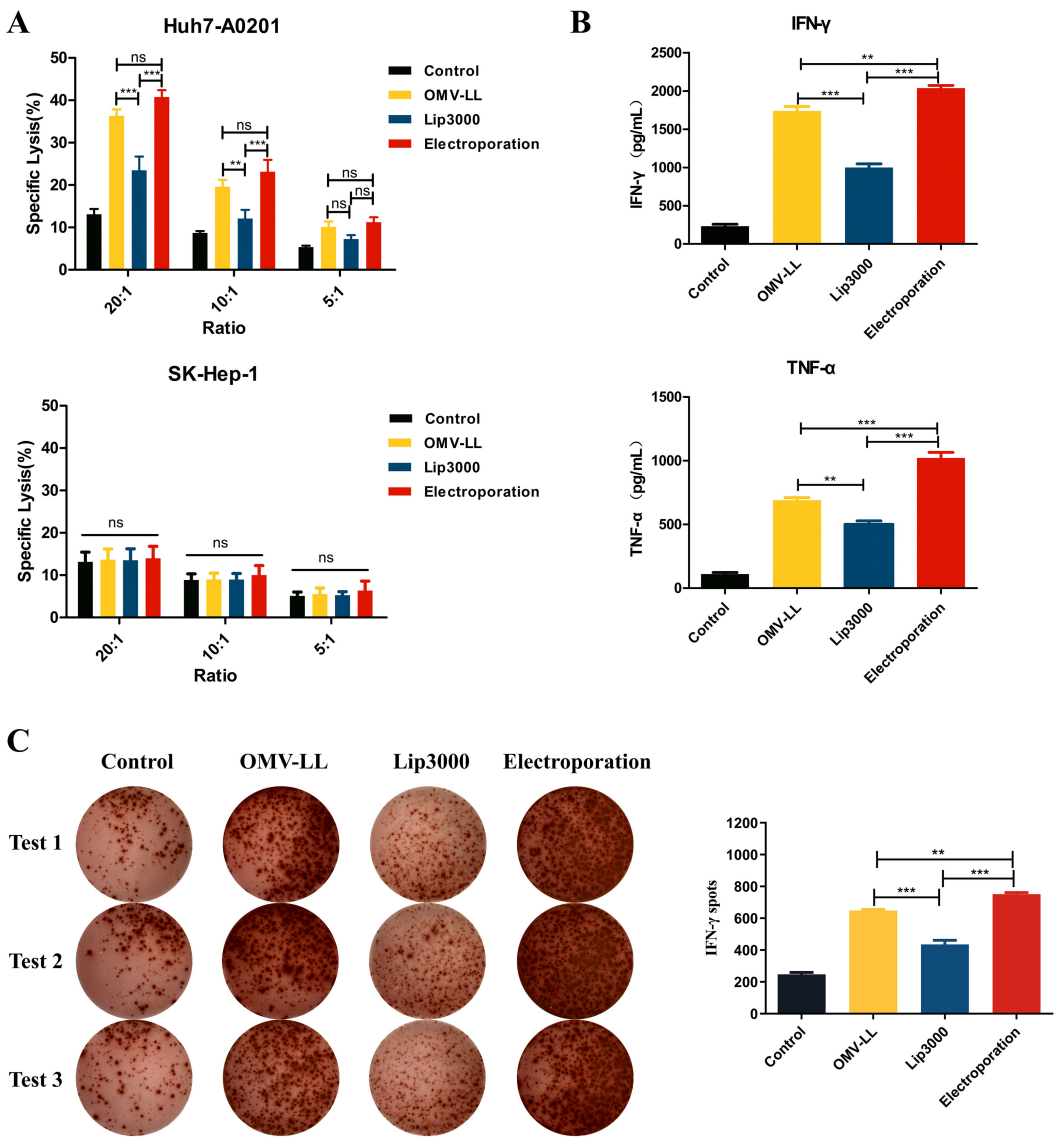
Killing effects of CTL induced in different ways on hepatocellular carcinoma *in vitro*. **(A)** LDH assay was performed to detect the killing effect of CTLs on target cells Huh7-A0201 and SK-Hep-1 at different potency-target ratios. **(B)** ELISA was performed to detect the killing of IFN-γ and TNF-α secreted by the target cells Huh7-A0201 by CTLs of each group under the condition of 10:1 potency-target ratio. **(C)** Under a 5:1 E-T ratio, ELISPOT detected IFN-γ^+^ cells during co-culture of CTL and target cells Huh7-A0201 in each group and did statistical analysis. (**P<0.01, ***P<0.001).

To evaluate the anti-hepatocellular carcinoma effects of CTLs induced by three different mRNA delivery methods, Huh7-A0201 and SK-Hep-1 cell lines were selected as target cells. LDH assays revealed that the cytotoxic activity of CTLs from the OMV-LL and electroporation groups against the target cells Huh7-A0201 at E:T ratios of 20:1 and 10:1 was notably higher than that of the Lip3000 group, and the OMV-LL group demonstrated the same cytotoxicity level as the electroporation group (**Figure 4A**). Meanwhile, no significant difference in the cytotoxicity against SK-Hep-1 cells was observed across all groups at the same E:T ratios (**Figure 4A**), suggesting that the CTLs’ cytotoxicity induced by mTP53_Y220C_ is antigen-specific.

Additionally, the secretion of pro-inflammatory cytokines IFN-γ and TNF-α was assessed by ELISA in each group of CTLs co-cultured with Huh7-A0201 at an E:T ratio of 10:1. The results indicated that the production of IFN-γ and TNF-α was significantly enhanced in both the OMV-LL and electroporation groups compared to the Lip3000 group. The OMV-LL group exhibited less levels of IFN-γ and TNF-α secretion than the electroporation group (**Figure 4B**). To further confirm the secretion of IFN-γ at the individual cell level, the ELISPOT method was used to identify IFN-γ^+^ CTLs. The results showed that both the OMV-LL and electroporation groups had a significantly greater number of IFN-γ^+^ CTLs compared to the Lip3000 group. The OMV-LL group also had a lower count of IFN-γ^+^ CTLs than the electroporation group (**Figure 4C**).

### 3.4 Anti-tumor ability of the CTLs induced by neoantigen mRNA *in vivo*

**Figure 5:**
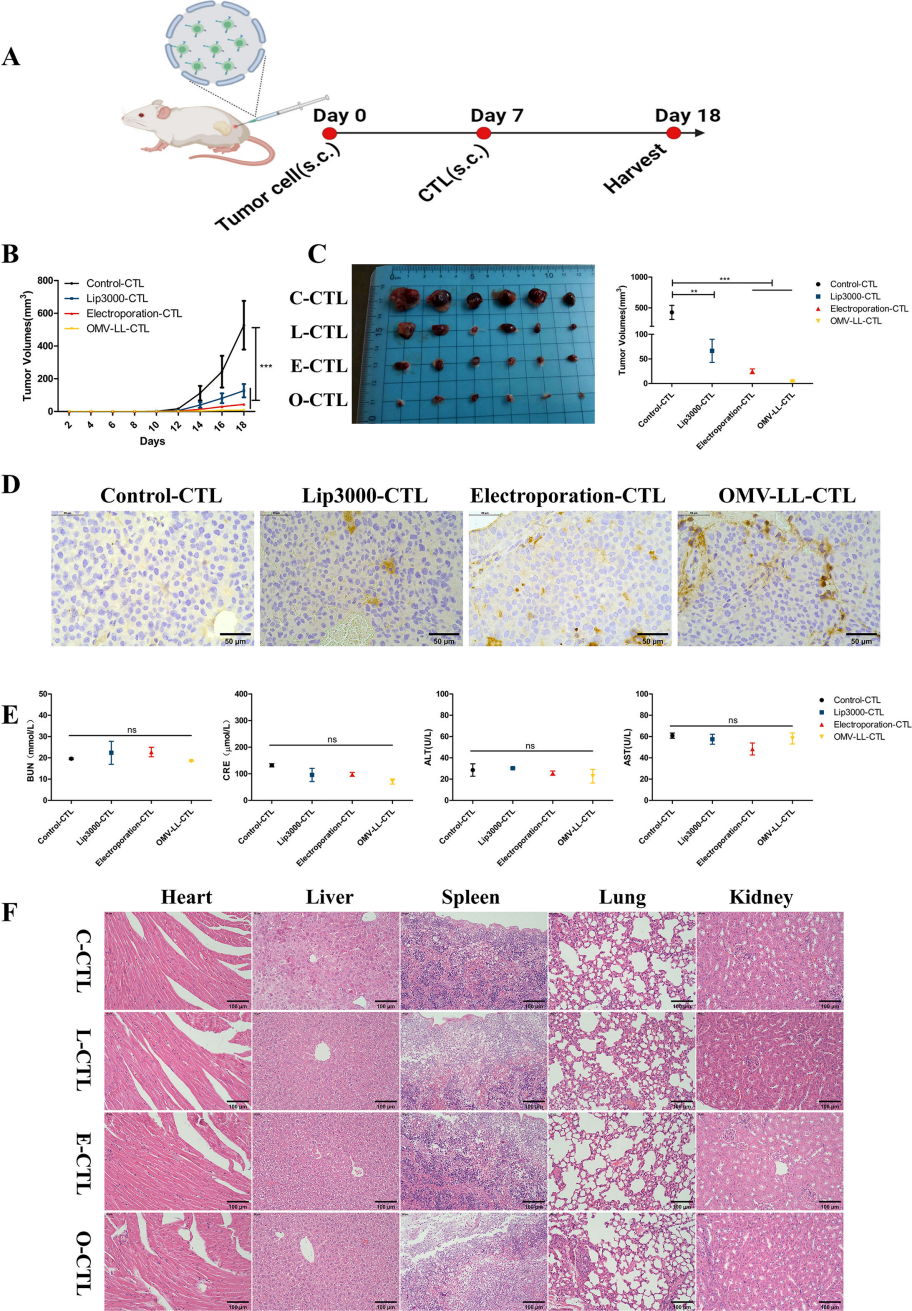
Specific CTLs inhibit hepatocellular carcinoma cell growth *in vivo*. **(A)**. Schematic diagram. **(B)** Tumor growth graph for each group of mice (n=6). **(C)** Tumor pictures and statistical graph of tumor size in each group. **(D)** Immunohistochemical detection of CD8^+^T-cell infiltration in each group of tumor sections (scale bar: 50 µm). **(E)** Biochemical analysis of serum BUN, CRE, ALT, and AST levels in tumor-bearing mice. **(F)** H&E images of organs including heart, liver, spleen, lung, and kidney (scale bar: 40µm). (**P<0.01, ***P<0.001).

In the *in vivo* study, a subcutaneous tumor model using Huh7-A0201 cells was established to assess the anti-tumor effects of CTLs activated by DCs transfected with Lip3000/mTP53_Y220C_, OMV-LL/mTP53_Y220C_, and electroporation/mTP53_Y220C_, respectively (**Figure 5A**). Compared to the control group, the average tumor growth curves in the OMV-LL, electroporation, and Lip3000 groups were slower, and the average tumor volumes showed a substantial decrease (**Figures 5B, C**). Analysis of tumor tissues revealed that infiltration of CD8^+^ T-cells was considerably higher in the OMV-LL and electroporation groups compared to the Control and Lip3000 groups (**Figure 5D**). These findings suggest that the anti-tumor effect of CTLs induced by DCs loaded with mTP53_Y220C_ was significantly more effective than that of CTLs not loaded with mTP53_Y220C_, and the ability of CTLs to infiltrate tumor tissue was markedly greater in the OMV-LL and electroporation groups compared to the Lip3000 group.

Eleven days after initiating CTL therapy, blood samples were collected from the mice to measure serum concentrations of blood urea nitrogen (BUN), creatinine (CRE), alanine transaminase (ALT), and aspartate transaminase (AST). The results indicated no notable differences in the BUN, CRE, ALT, and AST levels between the treated and control groups, suggesting that the CTL injections did not burden the mice’s liver and kidneys (**Figure 5E**). The main organs were also isolated and subjected to H&E staining, revealing no obvious pathological changes (**Figure 5F**). These results confirm that CTL therapy, administered via *in vivo* injection, exhibits good biosafety.

## 4 Discussion

DCs play a crucial role in antigen processing and presentation and in initiating immune responses to new antigens. When DCs take up mRNA coding for a neoantigen, it is translated into the corresponding protein, which is subsequently degraded by the proteasome within the DCs. The resulting peptide fragments are transported to the endoplasmic reticulum, where they bind to MHC class I molecules. These complexes are then presented on the surface of the dendritic cell, displaying the antigenic peptides alongside co-stimulatory signals to CD8^+^ T-cells, thus initiating the CTL response (23). Activation of T-cells requires three critical signals: (1) the binding of the peptide-major histocompatibility complex (p-MHC) to the T-cell receptor (2), the engagement of co-stimulatory molecules on antigen-presenting cells with their counterparts on T-cells, and (3) Cytokines secreted by APCs to T-cell activation (24, 25). In recent years, numerous studies have endeavored to boost the overall effectiveness of vaccines by amplifying these signals to enhance the communication between DCs and T-cells (26).

Our study evaluated three methods for delivering neoantigen mRNA: OMV-LL, electroporation, and Lip3000. Lip3000 is a standard mRNA delivery system in basic research. They can be readily synthesized and targeted to specific cell types by modifying their surface with specific ligands. However, the suboptimal delivery efficiency of Lip3000 and the potential to elicit only modest immune responses may limit the effectiveness of mRNA vaccines. Electroporation is a widely accepted process that facilitates the delivery of DNA/RNA in various kinds of cells by applying electrical pulses with appropriate amplitudes and waveforms in the special instrument. However, this technology has strict requirements on field intensity and the duration of the electrical pulse. Otherwise, it is easy to cause irreversible damage to the cell membrane and lead to cell lysis (27). Here, we found that the transfection efficiency of DCs via electroporation reached approximately 90%, significantly enhancing the presentation of neoantigens on the DC surface. Using a square wave instead of a conventional sinusoidal wave reduces the voltage to less than 400 V, decreasing cell death rates. It was interesting that although the transfection efficiency of OMV-LL is less than 10%, it has shown its ability to significantly increase the surface expression of co-stimulatory molecules CD80 and CD86 on DCs, enhancing CTL activation. OMVs are natural vesicles secreted by Gram-negative bacteria that can be effectively recognized and taken up by dendritic cells. In addition, OMVs are rich in pathogen-associated molecular patterns (PAMPs), which can strongly stimulate the innate immune system, promote antigen presentation, and activate T cells. Therefore, OMVs are ideal vaccine nanocarriers, as they can display and deliver exogenous antigens from microorganisms by fusing and expressing scaffold proteins on the OMV surface. Previous studies have reported that the OMV-LL as an mRNA vaccine delivery carrier, can significantly inhibit the progression of melanoma in mice and lead to complete tumour regression in 37.5% mouse models of colorectal cancer, even induce long-term immune memory, continuing to protect mice from tumour attacks 60 days later (19). OMV-LL contains unique prokaryotic components, including peptidoglycan, cytosolic acid, and phosphatidic acid, recognized by various pattern recognition receptors, such as Toll 1, Toll 2, and Toll 4. The pathogen-associated molecular patterns within OMV-LL activate various innate immune signaling pathways, generating a natural adjuvant effect that upregulates the expression of CD80 and CD86 on the DC surface. This, in turn, influences CD28 on the T-cell surface, regulating T-cell activation, cytokine expression, and antigen-specific CTL production (28–30). As stated in the three-signal theory, the first signal in the electroporation group is the strongest, but the second and third signals are weaker than those in the OMV-LL and Lip3000 groups, ultimately resulting in T cell activation levels in the OMV-LL group being comparable to those in the electroporation group.

Although electroporation has high delivery efficiency and can effectively deliver neoantigens, its high cell death rate of up to 50% limits its large-scale application. In contrast, while OMV-LL has lower efficiency in delivering neoantigens, its natural immune adjuvant effect amplifies the effect, making its T cell activation efficacy comparable to that of electroporation. Considering clinical translation, OMV-LL amplification is simple and rapid, making it suitable not only as an mRNA vaccine carrier but also for *in vitro* stimulation of DC vaccines. The electroporation technique is limited by the cell survival rate and the electroporation equipment, and currently its application scale is also restricted to some extent. OMVs have been demonstrated clinical potential and successfully used in OMV-based vaccines against Neisseria meningitidis in multiple clinical trials, showing strong immunogenicity and safety, leading to their approval for preventing epidemic meningitis (31). Furthermore, the application of OMVs in cancer immunotherapy is being explored to deliver tumor antigens and elicit specific immune responses (32). Preclinical studies have shown that engineered OMVs can deliver siRNA or CRISPR/Cas systems effectively (33, 34), inhibiting tumor growth or correcting genetic mutations, paving the way for future clinical applications. Despite this, the efficiency of OMV-LL loading neoantigen mRNA was lower than that of electroporation in our results, which needs further improvement. Here, the mRNA was externally loaded on OMV-LL and might be easily stripped. OMV-LL could also carry a large amount of nuclease, accelerating mRNA degradation. On the other hand, OMVs released from some pathogenic bacteria may carry disease-causing agents (35, 36), presenting challenges such as heterogeneity, standardization, and potential pathogenicity that must be addressed in vaccine development to ensure their safety and efficacy. Therefore, in subsequent studies, in addition to inhibiting nuclease in the OMV-LL system, we considered loading mRNA into the interior of OMV, which could reduce the effect of liquid flow shear during *in vivo* delivery of OMV to maximize the efficacy of this mRNA vaccine.

Individuals with late-stage cancers often show minimal responses to single-agent immunotherapies, leading to an increased interest in combination immunotherapeutic approaches. Elevated PD-L1 expression has been noted during treatment with mRNA-based personalized vaccines (37). PD-L1 is usually constitutively expressed in most cancers, but inducible expression is more prevalent in HCC. IFN-γ, a significant factor released by tumor-infiltrating CD8^+^ T-cells, is known to upregulate PD-L1 expression on HCC cells (38). In this study, DC-induced CTLs loaded with mTP53_Y220C_ secreted more IFN-γ, enhancing their ability to kill target cells. However, IFN-γ boosts anti-tumor effects and increases PD-L1 levels on HCC cells (39, 40), potentially dampening the overall immune response. Research indicates that lower surface levels of PD-L1 are more conducive to effective antigen presentation and activation of CTLs (41). Future studies should investigate the anti-tumor effects of combining immune checkpoint blockade therapy with CTL strategies to potentially counteract the PD-L1 upregulation induced by IFN-γ.

## Data Availability

The raw data supporting the conclusions of this article will be made available by the authors, without undue reservation.

## References

[1] Xia C Dong X Li H Cao M Sun D He S. Cancer statistics in China and United States, 2022: profiles, trends, and determinants. Chin Med J. (2022) 135:584–90. doi: 10.1097/CM9.0000000000002108, PMID: 35143424 PMC8920425

[2] Saxena M van der Burg SH Melief CJM Bhardwaj N. Therapeutic cancer vaccines. Nat Rev Cancer. (2021) 21:360–78. doi: 10.1038/s41568-021-00346-0, PMID: 33907315

[3] Rojas LA Sethna Z Soares KC Olcese C Pang N Patterson E. Personalized RNA neoantigen vaccines stimulate T cells in pancreatic cancer. Nature. (2023) 618:144–50. doi: 10.1038/s41586-023-06063-y, PMID: 37165196 PMC10171177

[4] Ott PA Hu-Lieskovan S Chmielowski B Govindan R Naing A Bhardwaj N. A phase ib trial of personalized neoantigen therapy plus anti-PD-1 in patients with advanced melanoma, non-small cell lung cancer, or bladder cancer. Cell. (2020) 183:347–62.e24. doi: 10.1016/j.cell.2020.08.053, PMID: 33064988

[5] Xie N Shen G Gao W Huang Z Huang C Fu L. Neoantigens: promising targets for cancer therapy. Signal transduction targeted Ther. (2023) 8:9. doi: 10.1038/s41392-022-01270-x, PMID: 36604431 PMC9816309

[6] Shah RK Cygan E Kozlik T Colina A Zamora AE. Utilizing immunogenomic approaches to prioritize targetable neoantigens for personalized cancer immunotherapy. Front Immunol. (2023) 14:1301100. doi: 10.3389/fimmu.2023.1301100, PMID: 38149253 PMC10749952

[7] Chasov V Zaripov M Mirgayazova R Khadiullina R Zmievskaya E Ganeeva I. Promising new tools for targeting p53 mutant cancers: humoral and cell-based immunotherapies. Front Immunol. (2021) 12:707734. doi: 10.3389/fimmu.2021.707734, PMID: 34484205 PMC8411701

[8] Kastenhuber ER Lowe SW. Putting p53 in context. Cell. (2017) 170:1062–78. doi: 10.1016/j.cell.2017.08.028, PMID: 28886379 PMC5743327

[9] Wang L Yan K He X Zhu H Song J Chen S. LRP1B or TP53 mutations are associated with higher tumor mutational burden and worse survival in hepatocellular carcinoma. J Cancer. (2021) 12:217–23. doi: 10.7150/jca.48983, PMID: 33391418 PMC7738815

[10] Yang H Sun L Guan A Yin H Liu M Mao X. Unique TP53 neoantigen and the immune microenvironment in long-term survivors of Hepatocellular carcinoma. Cancer immunology immunotherapy: CII. (2021) 70:667–77. doi: 10.1007/s00262-020-02711-8, PMID: 32876735 PMC10992148

[11] Miao L Zhang Y Huang L. mRNA vaccine for cancer immunotherapy. Mol cancer. (2021) 20:41. doi: 10.1186/s12943-021-01335-5, PMID: 33632261 PMC7905014

[12] Jia L Mao Y Ji Q Dersh D Yewdell JW Qian SB. Decoding mRNA translatability and stability from the 5’ UTR. Nat Struct Mol Biol. (2020) 27:814–21. doi: 10.1038/s41594-020-0465-x, PMID: 32719458

[13] Passmore LA Coller J. Roles of mRNA poly(A) tails in regulation of eukaryotic gene expression. Nat Rev Mol Cell Biol. (2022) 23:93–106. doi: 10.1038/s41580-021-00417-y, PMID: 34594027 PMC7614307

[14] Wei Z Zhang S Wang X Xue Y Dang S Zhai J. Technological breakthroughs and advancements in the application of mRNA vaccines: a comprehensive exploration and future prospects. Front Immunol. (2025) 16:1524317. doi: 10.3389/fimmu.2025.1524317, PMID: 40103818 PMC11913674

[15] Kuzmin IV Soto Acosta R Pruitt L Wasdin PT Kedarinath K Hernandez KR. Comparison of uridine and N1-methylpseudouridine mRNA platforms in development of an Andes virus vaccine. Nat Commun. (2024) 15:6421. doi: 10.1038/s41467-024-50774-3, PMID: 39080316 PMC11289437

[16] Kranz LM Diken M Haas H Kreiter S Loquai C Reuter KC. Systemic RNA delivery to dendritic cells exploits antiviral defence for cancer immunotherapy. Nature. (2016) 534:396–401. doi: 10.1038/nature18300, PMID: 27281205

[17] De Keersmaecker B Claerhout S Carrasco J Bar I Corthals J Wilgenhof S. TriMix and tumor antigen mRNA electroporated dendritic cell vaccination plus ipilimumab: link between T-cell activation and clinical responses in advanced melanoma. J immunotherapy Cancer. (2020) 8:e000329. doi: 10.1136/jitc-2019-000329, PMID: 32114500 PMC7057443

[18] Surana R Lebleu V Lee J Smaglo B Zhao D Lee M. Phase I study of mesenchymal stem cell (MSC)-derived exosomes with KRAS G12D siRNA in patients with metastatic pancreatic cancer harboring a KRAS G12D mutation. J Clin Oncol. (2022) 40:TPS633–TPS. doi: 10.1200/JCO.2022.40.4_suppl.TPS633

[19] Li Y Ma X Yue Y Zhang K Cheng K Feng Q. Rapid surface display of mRNA antigens by bacteria-derived outer membrane vesicles for a personalized tumor vaccine. Advanced materials (Deerfield Beach Fla). (2022) 34:e2109984. doi: 10.1002/adma.202109984, PMID: 35315546

[20] Mayakonda A Lin DC Assenov Y Plass C Koeffler HP. Maftools: efficient and comprehensive analysis of somatic variants in cancer. Genome Res. (2018) 28:1747–56. doi: 10.1101/gr.239244.118, PMID: 30341162 PMC6211645

[21] Malekzadeh P Pasetto A Robbins PF Parkhurst MR Paria BC Jia L. Neoantigen screening identifies broad TP53 mutant immunogenicity in patients with epithelial cancers. J Clin Invest. (2019) 129:1109–14. doi: 10.1172/JCI123791, PMID: 30714987 PMC6391139

[22] Chen F Zou Z Du J Su S Shao J Meng F. Neoantigen identification strategies enable personalized immunotherapy in refractory solid tumors. J Clin Invest. (2019) 129:2056–70. doi: 10.1172/JCI99538, PMID: 30835255 PMC6486339

[23] Jhunjhunwala S Hammer C Delamarre L. Antigen presentation in cancer: insights into tumour immunogenicity and immune evasion. Nat Rev Cancer. (2021) 21:298–312. doi: 10.1038/s41568-021-00339-z, PMID: 33750922

[24] Edner NM Carlesso G Rush JS Walker LSK. Targeting co-stimulatory molecules in autoimmune disease. Nat Rev Drug discovery. (2020) 19:860–83. doi: 10.1038/s41573-020-0081-9, PMID: 32939077

[25] Curtsinger JM Lins DC Mescher MF. Signal 3 determines tolerance versus full activation of naive CD8 T cells: dissociating proliferation and development of effector function. J Exp Med. (2003) 197:1141–51. doi: 10.1084/jem.20021910, PMID: 12732656 PMC2193970

[26] You Y Jin F Du Y Zhu L Liu D Zhu M. A photo-activable nano-agonist for the two-signal model of T cell *in vivo* activation. J Controlled release. (2023) 361:681–93. doi: 10.1016/j.jconrel.2023.08.033, PMID: 37595667

[27] Choi SE Khoo H Hur SC. Recent advances in microscale electroporation. Chem Rev. (2022) 122:11247–86. doi: 10.1021/acs.chemrev.1c00677, PMID: 35737882 PMC11111111

[28] Gilmore WJ Johnston EL Bitto NJ Zavan L O’Brien-Simpson N Hill AF. Bacteroides fragilis outer membrane vesicles preferentially activate innate immune receptors compared to their parent bacteria. Front Immunol. (2022) 13:970725. doi: 10.3389/fimmu.2022.970725, PMID: 36304461 PMC9592552

[29] Gilmore WJ Johnston EL Zavan L Bitto NJ Kaparakis-Liaskos M. Immunomodulatory roles and novel applications of bacterial membrane vesicles. Mol Immunol. (2021) 134:72–85. doi: 10.1016/j.molimm.2021.02.027, PMID: 33725501

[30] Bitto NJ Cheng L Johnston EL Pathirana R Phan TK Poon IKH. Staphylococcus aureus membrane vesicles contain immunostimulatory DNA, RNA and peptidoglycan that activate innate immune receptors and induce autophagy. J extracellular vesicles. (2021) 10:e12080. doi: 10.1002/jev2.12080, PMID: 33815695 PMC8015888

[31] Micoli F MacLennan CA. Outer membrane vesicle vaccines. Semin Immunol. (2020) 50:101433. doi: 10.1016/j.smim.2020.101433, PMID: 33309166

[32] Zhao X Zhao R Nie G. Nanocarriers based on bacterial membrane materials for cancer vaccine delivery. Nat Protoc. (2022) 17:2240–74. doi: 10.1038/s41596-022-00713-7, PMID: 35879454

[33] Zhou X Miao Y Wang Y He S Guo L Mao J. Tumour-derived extracellular vesicle membrane hybrid lipid nanovesicles enhance siRNA delivery by tumour-homing and intracellular freeway transportation. J extracellular vesicles. (2022) 11:e12198. doi: 10.1002/jev2.12198, PMID: 35233952 PMC8888792

[34] Yao X Lyu P Yoo K Yadav MK Singh R Atala A. Engineered extracellular vesicles as versatile ribonucleoprotein delivery vehicles for efficient and safe CRISPR genome editing. J extracellular vesicles. (2021) 10:e12076. doi: 10.1002/jev2.12076, PMID: 33747370 PMC7962171

[35] Rueter C Bielaszewska M. Secretion and Delivery of Intestinal Pathogenic Escherichia coli Virulence Factors via Outer Membrane Vesicles. Front Cell infection Microbiol. (2020) 10:91. doi: 10.3389/fcimb.2020.00091, PMID: 32211344 PMC7068151

[36] Chen S Lei Q Zou X Ma D. The role and mechanisms of gram-negative bacterial outer membrane vesicles in inflammatory diseases. Front Immunol. (2023) 14:1157813. doi: 10.3389/fimmu.2023.1157813, PMID: 37398647 PMC10313905

[37] Sayour EJ Grippin A De Leon G Stover B Rahman M Karachi A. Personalized tumor RNA loaded lipid-nanoparticles prime the systemic and intratumoral milieu for response to cancer immunotherapy. Nano letters. (2018) 18:6195–206. doi: 10.1021/acs.nanolett.8b02179, PMID: 30259750 PMC6597257

[38] Zou J Zhuang M Yu X Li N Mao R Wang Z. MYC inhibition increases PD-L1 expression induced by IFN-γ in hepatocellular carcinoma cells. Mol Immunol. (2018) 101:203–9. doi: 10.1016/j.molimm.2018.07.006, PMID: 30007230

[39] Moon JW Kong SK Kim BS Kim HJ Lim H Noh K. IFNγ induces PD-L1 overexpression by JAK2/STAT1/IRF-1 signaling in EBV-positive gastric carcinoma. Sci Rep. (2017) 7:17810. doi: 10.1038/s41598-017-18132-0, PMID: 29259270 PMC5736657

[40] Knopf P Stowbur D Hoffmann SHL Hermann N Maurer A Bucher V. Acidosis-mediated increase in IFN-γ-induced PD-L1 expression on cancer cells as an immune escape mechanism in solid tumors. Mol cancer. (2023) 22:207. doi: 10.1186/s12943-023-01900-0, PMID: 38102680 PMC10722725

[41] Okada M Shimizu K Iyoda T Ueda S Shinga J Mochizuki Y. PD-L1 expression affects neoantigen presentation. iScience. (2020) 23:101238. doi: 10.1016/j.isci.2020.101238, PMID: 32629606 PMC7322261

